# On Therapeutic Plasma Exchange Against Severe COVID-19-Associated Pneumonia: An Observational Clinical Study

**DOI:** 10.3389/fnut.2022.809823

**Published:** 2022-02-22

**Authors:** Luca Cegolon, Behzad Einollahi, Yunes Panahi, Sina Imanizadeh, Mohammad Rezapour, Mohammad Javanbakht, Mohammad Nikpouraghdam, Hassan Abolghasemi, Giuseppe Mastrangelo

**Affiliations:** ^1^Department of Medical, Surgical and Health Sciences, University of Trieste, Trieste, Italy; ^2^Public Health Department, University Health Agency Giuliano-Isontina (ASUGI), Trieste, Italy; ^3^Nephrology and Urology Research Center, Baqiyatallah University of Medical Sciences, Tehran, Iran; ^4^Pharmacotherapy Department, Faculty of Pharmacy, Baqiyatallah University of Medical Sciences, Tehran, Iran; ^5^Student Research Committee (SRC), Baqiyatallah University of Medical Sciences, Tehran, Iran; ^6^Applied Microbiology Research Center, Systems Biology and Poisonings Institute, Baqiyatallah University of Medical Sciences, Tehran, Iran; ^7^Department of Cardiac, Thoracic, Vascular Sciences and Public Health, Padua University, Padua, Italy

**Keywords:** COVID-19, cytokine storm, therapeutic plasma exchange—TPE, Plasmapheresis (PP), pneumonia—clinical features and management

## Abstract

**Background:**

There is a risk of novel mutations of SARS-CoV-2 that may render COVID-19 resistant to most of the therapies, including antiviral drugs and vaccines. The evidence around the application of therapeutic plasma exchange (TPE) for the management of critically ill patients with COVID-19 is still provisional, and further investigations are needed to confirm its eventual beneficial effects.

**Aims:**

To assess the effect of TPE on the risk of mortality in patients with COVID-19-associated pneumonia, using three statistical procedures to rule out any threats to validity.

**Methods:**

We therefore carried out a single-centered retrospective observational non-placebo-controlled trial enrolling 73 inpatients from Baqiyatallah Hospital in Tehran (Iran) with the diagnosis of COVID-19-associated pneumonia confirmed by real-time polymerase chain reaction (RT-qPCR) on nasopharyngeal swabs and high-resolution computerized tomography chest scan. These patients were broken down into two groups: Group 1 (30 patients) receiving standard care (corticosteroids, ceftriaxone, azithromycin, pantoprazole, hydroxychloroquine, lopinavir/ritonavir), and Group 2 (43 patients) receiving the above regimen plus TPE (replacing 2 l of patients' plasma by a solution, 50% of normal plasma, and 50% of albumin at 5%) administered according to various time schedules. The follow-up time was 30 days and all-cause mortality was the endpoint.

**Results:**

Deaths were 6 (14%) in Group 2 and 14 (47%) in Group 1. However, different harmful risk factors prevailed among patients not receiving TPE rather than being equally split between the intervention and control group. We used an algorithm of structural equation modeling (of STATA) to summarize a large pool of potential confounders into a single score (called with the descriptive name “severity”). Disease severity was lower (Wilkinson rank-sum test *p* < 0.001) among patients with COVID-19 undergoing TPE (median: −2.82; range: −5.18; 7.96) as compared to those not receiving TPE (median: −1.35; range: −3.89; 8.84), confirming that treatment assignment involved a selection bias of patients according to the severity of COVID-19 at hospital admission. The adjustment for confounding was carried out using severity as the covariate in Cox regression models. The univariate hazard ratio (HR) of 0.68 (95%CI: 0.26; 1.80; *p* = 0.441) for TPE turned to 1.19 (95%CI: 0.43; 3.29; *p* = 0.741) after adjusting for severity.

**Conclusions:**

In this study sample, the lower mortality observed among patients receiving TPE was due to a lower severity of COVID-19 rather than the TPE effects.

## Introduction

COVID-19 is an asymptomatic disease in most cases, but some patients develop life-threatening diseases characterized by acute respiratory distress syndrome (ARDS), sepsis, multisystem organ failure (MOF), extrapulmonary manifestations, thromboembolic disease, and associated cytokine release syndrome (CRS) (1–3). Although the pathophysiology of COVID-19 is far from being completely understood, the severe form of the disease is correlated with overrelease of proinflammatory cytokines (tumor necrosis factor, IL-6, and IL-1β), which cause strong inflammation, endothelial injury, thrombotic microangiopathy, MOF, and eventually death (4–6). The strict biological criteria to diagnose the CRS associated with COVID-19 remain however poorly defined (7).

The lack of effective treatments against COVID-19 leads to a sense of urgency to develop new therapeutic strategies based on pathophysiological assumptions, thus endorsing the hypothesis that properly timed antiinflammatory therapeutic strategies could improve patients' clinical outcomes and prognosis (6, 8). The mortality risk associated with the above CRS is thought to increase with the persistence of high blood concentration of cytokines over time; hence some therapeutic strategies against critical COVID-19 are focusing on anticytokine treatments or immunomodulators (6, 9).

A non-pharmacological option to counteract the dysregulated proinflammatory response featuring severe COVID-19 could be represented by blood purification techniques (8). Therapeutic plasma exchange (TPE) is an extracorporeal treatment performed by filtrating a volume of plasma equivalent to the estimated plasma volume that selectively removes circulating pathogenetic substances, such as autoreactive antibodies, immune complexes, paraproteins, lipoproteins, and inflammatory mediators like cytokines. TPE has been applied to manage different critical diseases, including the acute respiratory distress syndrome (ARDS) (10), pneumonia and respiratory failure from H1N1 influenza A virus (11), Kawasaki disease (12), and sepsis, effectively reducing the elevated levels of cytokines and inflammatory mediators, avoiding lethal complications as septic shock, pulmonary embolism, renal injury, or disseminated intravascular coagulation (2, 10, 12–14).

Therapeutic plasma exchange as a remedy for the cytokine storm has also already been used as a supportive treatment for critically ill patients with COVID-19 (2, 10), especially among those admitted to intensive care unit (ICU) (6). The risk of novel mutations rendering SARS-CoV-2 resistant to most therapies (including vaccines and antiviral drugs) might reduce the spectrum of drugs available for COVID-19. TPE, which has been performed for over a century, has proved to be safe and effective in several disorders (15). Overall, TPE can be considered as a salvage or adjunctive treatment against severe COVID-19, with the rationale of clearing out the related cytokine storm and possibly the viral load (16).

However, the effect of TPE has not been studied in patients infected with SARS-CoV-1 and MERS. Moreover, TPE does not appear in the Coronavirus Disease 2019 (COVID-19) Treatment Guidelines (major revisions on March 5, 2021 and February 23, 2021) issued by the National Institutes of Health (17). Although limited case reports demonstrated a beneficial effect, the evidence on the respective efficacy against COVID-19 is still inconclusive (18).

### Aims

To assess the effect of TPE on the risk of mortality in patients with COVID-19-associated pneumonia using three statistical procedures to rule out the possibility that any threats to internal validity could be responsible for the observed treatment effect.

## Materials and Methods

### Study Design

This single-centered retrospective observational controlled, yet not randomized, study enrolled 73 inpatients from Baqiyatallah Hospital in Tehran (Iran) between March 4 and May 20, 2020. All patients with respiratory symptoms were screened by clinical examination, real-time PCR (RT-qPCR) on nasopharyngeal swabs, and chest-computerized tomography (CT) scan to confirm the diagnosis of COVID-19-associated pneumonia.

### Clinical Data Collection

The following inclusion criteria were applied to recruit patients with COVID-19 for this study:

• age ≥ 20 years;
• severe pneumonia with lung infiltrates on more than 50% of the lung fields and evidence of hypoxiemia, featured by:
  - tachypnoea (≥ 30 breaths per min);
  - O_2_ saturation (SpO_2_) ≤ 90% at rest on room air; or
  - PaO_2_/FiO_2_ ratio <300 mm Hg);
• positive real-time RT-qPCR (throat-swab specimens) or typical COVID-19-associated pneumonia imaging at chest CT scan, following WHO interim guidelines: evidence of severe pneumonia, patchy infiltration, ground glass opacities, ill-defined margins, smooth or irregular interlobular septal thickening, air bronchogram, crazy-paving pattern, and thickening of the adjacent pleura with severe involvement (> 50%).

The following exclusion criteria were applied (19):

- non-severe COVID-19 patients,
- MOF in COVID-19 patients.

Patients' demographic and health data were extracted from the medical records. All medical records were screened by a double opinion of two hospital doctors. Adjudication of any clinical interpretative diagnostic difference was performed by a pulmonologist. Missing clinical data were filled up after discussion with an health care staff.

**Figure 1:**
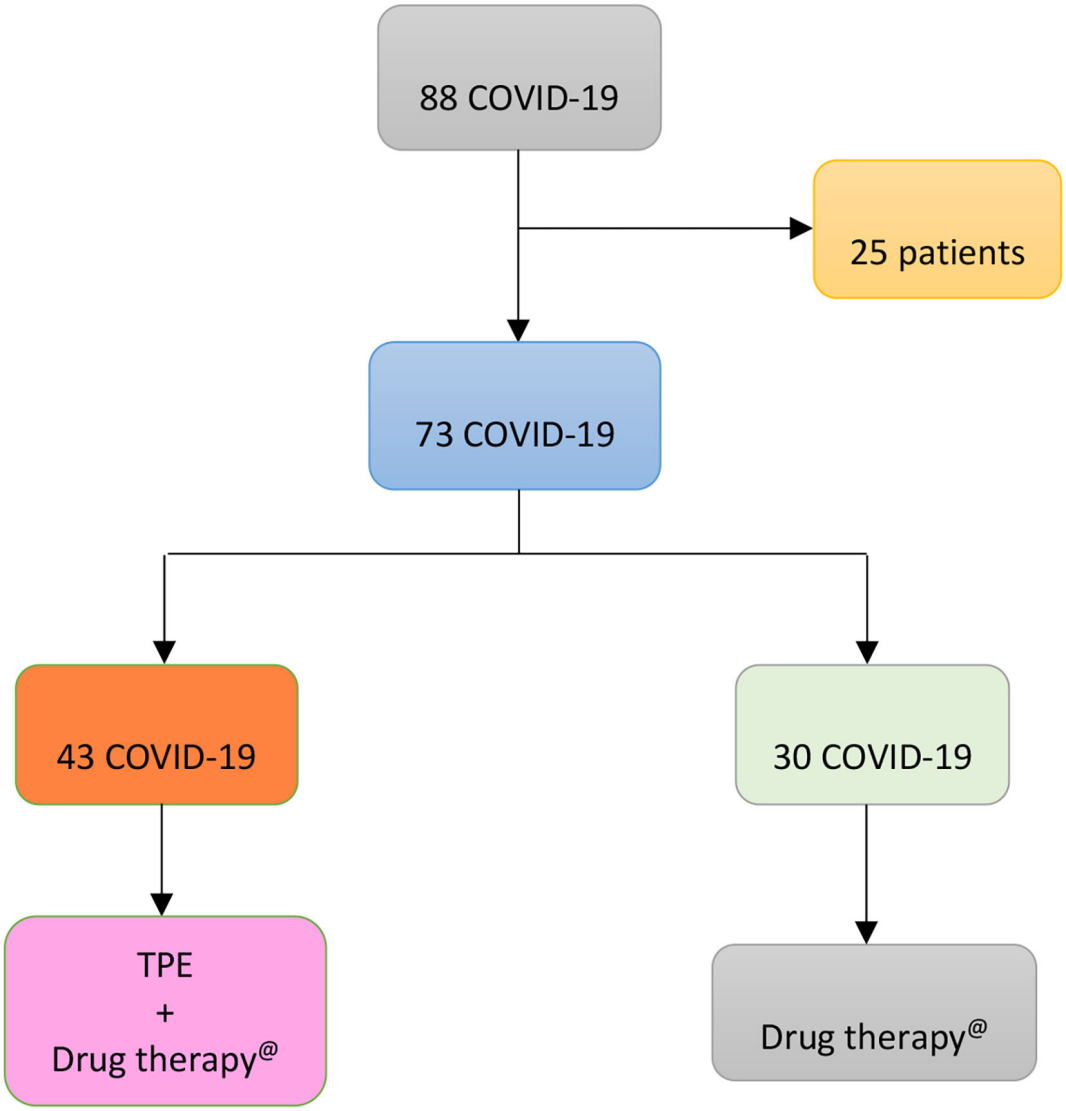
Flow chart displaying the apportionment of 88 COVID-19 patients admitted to Baqiyatallah Hospital in Tehran (Iran) between 24 March and 20 May 2020. 25 patients were excluded because of multi-organ failure and 73 patients included in the study. The latter were broken down, according to clinical decision of doctors and patients' will, in two groups: 30 patients (Group 1) untreated and 43 patients (Group 2) treated with TPE.

According to clinical decision of doctors and patients' will, patients with COVID-19 were broken down as shown in Figure 1:

- **Group 1** (30 patients) receiving high doses of corticosteroids (methylprednisolone i.v. pulse, single daily dose of 500 mg on day 1, 250 mg on day 2 and day 3), ceftriaxone (1 gr twice a day, oral), azithromycin (500 mg/daily, oral) pantoprazole (40 mg twice a day, oral), hydroxychloroquine (200 mg/12 h, oral), and lopinavir/ritonavir (200 mg/50 mg daily, oral tablets).
- **Group 2** (43 patients) receiving the above regimen plus TPE, replacing 2 L of patients' plasma by 1 L of fresh frozen plasma (FFP) and 1 L of a 5% albumin solution.

### Ethical Statement

This study was approved by the Ethical Committee of Baqiyatallah University of Medical Sciences (IR.BMSU.REC.1398435; IRCT registration number: IRCT20080901001165N58; Registration date: 2020-05-27) (19). All ethical guidelines for studies on human subjects were carefully observed and informed consent was obtained from study participants.

### Variables

#### Risk Factors

According to the Centers for Disease Control and Prevention (CDC) (20), older age, chronic obstructive pulmonary disease, cardiovascular disease, type 2 diabetes mellitus, obesity, sickle cell disease, chronic kidney disease, immunocompromised status, and cancer are risk factors for severe COVID-19 (20). We considered these factors if they were actually found in the clinical records of the patients. We also included in the statistical analysis additional conditions for which the data were unclear: comorbidity (grouping miscellaneous conditions), male sex (although is not currently included in the CDC list of risk factors for COVID-19), type 1 diabetes mellitus, hypertension, and smoking. All the above risk factors were treated as dichotomous variables in the statistical analysis.

#### Criteria of Classification

In the present study, patients with COVID-19 were categorized according to vital status or by TPE administration. For every single risk factor, the risk of death, and/or the probability of being assigned to TPE was estimated.

#### Oxygen Support

The disease is a severe pneumonia, limiting gas exchange of lung. Rather than chest CT scan imaging, we assumed as an indicator of lung involvement the heaviest O_2_ delivery support ever administered to patients. The variable was categorized as 0 (high-flow nasal canula), 1 (non-invasive mechanical ventilation) and 2 (invasive mechanical ventilation with intubation). The variable was treated as ordered polytomous variable in the analysis.

#### TPE Administration

Early initiation, duration, and quantity of TPE could be related to better outcomes. Hence, TPE administration was categorized according to days, ranging from 1 to 12, of treatment start since hospital admission, coding a new variable (timing) as follows:

- 0 (sample including the above Group 1);
- 1 (patients admitted to TPE on days 1 to 3);
- 2 (patients treated on days 4 to 5); and
- 3 (patients admitted to TPE 6–12 days since hospital admission).

We also coded a variable (number of treatments) with 3 levels:

- 0 (including the above Group 1),
- 1 (patients pertaining to Group 2 who underwent 1 to 4 sessions of TPE); and
- 2 (Group 2 patients with 5 TPEs).

#### The Latent Variable Severity

The latter term is not an observed variable but is estimated by SEM, as a single score summarizing a large number of measured pretreatment covariates, particularly useful to adjust for confounders using Cox regression models (see below).

### Descriptive Analysis

The risk factors of 73 COVID-19 patients, broken down by vital status or TPE treatment, were reported in rows and columns of Table 1 to summarize the relationships among observations. At each row and column interception, there were numbers and percentages of subjects having a given trait; the denominator of the percentage was always 53 for patients who survived, 20 for those deceased, 43 for patients treated with TPE, and 30 for those not undergoing TPE (“Total” in the last row of Table 1). Risk factors for severe COVID-19 were mainly dichotomous variables (e.g., sex). Table 1 reports only one of the two possible values, the complementary one being easily calculated by subtraction using the total figures (numbers) or 1.00 (percentages). Conversely, all the possible categories of polytomous variables (for example, O_2_ support) are reported in Table 1. Besides numbers and percentages, Table 1 displays the odds ratios (OR), estimated with an exact method due to the relatively limited number of study subjects, with the respective 95% confidence interval (95%CI) and the two-tail *p*-value. By default, the conditional maximum likelihood estimates were used in the OR estimation, except for those parameters (e.g., ICU admission) for which a percentage was equal to 100% and the upper bound of 95%CI was infinite. In such a case OR was obtained by median unbiased estimates. OR is a measure of association between an exposure and an outcome. The OR represents the odds that an outcome will occur given a particular exposure, compared to the odds of the outcome occurring in the absence of that exposure. The outcome was “death” in the analysis for columns 2 and 3, or “TPE treatment” for columns 5 and 6. The multiple categories of polytomous variables were coded as ordinal variables (0, 1, 2, etc.), but were considered as “continuous variables” in the context of exact logistic regression analysis; therefore, only one OR was returned by the statistical program.

**Table 1:** Distribution of 73 patients with COVID-19 by vital and treatment status: number (*N*) and percentage (%) of cases; exact odds ratio (OR); 95% confidence interval (95%CI) and two-tail *p*-values.

**FACTORS**	**Distribution by vital status**				**Distribution by treatment status**		
		**Deceased *N* (%)**	**Survived *N* (%)**	**OR** **(95%CI)** ***p*-value**	**TPE *N* (%)**	**No TPE *N* (%)**	**OR** **(95%CI)** ***p*-value**
O_2_ support	High-flow nasal canula	1 (5)	18 (34)	89.4	14 (32)	5 (17)	0.47
	Non-invasive ventilation	3 (15)	35 (66)	(11.2; 4371.0)	23 (54)	15 (50)	(0.21; 0.996)
	Invasive ventilation	16 (80)	0 (0)	<0.001	6 (14)	10 (33)	0.049
ICU admission		20 (100)	20 (38)	43.5 (6.92; +Inf) <0.001	20 (47)	20 (67)	0.44 (0.15; 1.26) 0.142
65+ years of age		12 (60)	12 (22)	4.99 (1.50; 17.9) 0.007	12 (28)	12 (40)	0.59 (0.19; 1.76) 0.407
20+ days of hospitalization		10 (50)	10 (19)	4.20 (1.22; 15.1) 0.021	7 (16)	13 (43)	0.26 (0.07; 0.85) 0.023
Diabetes		9 (45)	15 (28)	2.05 (0.61; 6.79) 0.283	11 (26)	13 (43)	0.45 (0.15; 1.36) 0.183
Hypertension		11 (55)	20 (38)	2.00 (0.63; 6.54) 0.287	16 (37)	15 (50)	0.60 (0.21; 1.69) 0.397
Comorbidities		10 (50)	24 (45)	1.21 (0.38; 3.84) 0.921	18 (42)	16 (53)	0.63 (0.22; 1.78) 0.466
Male sex		10 (50)	31 (58)	0.71 (0.22; 2.27) 0.6958	29 (67)	12 (40)	3.06 (1.06; 9.18) 0.037
Ex/current smokers		1 (5)	4 (8)	0.65 (0.01; 7.12) 1.000	4 (9)	1 (3)	2.94 (0.27; 1.51) 0.621
Plasmapheresis (TPE)		6 (30)	37 (70)	0.19 (0.05; 0.64) 0.005	43 (100)	NA	NA
Number of treatments	0	14 (70)	16 (30)	0.37	NA	30 (100)	NA
	1	3 (15)	14 (26)	(0.16; 0.76)	17 (40)	NA	NA
	2	3 (15)	23 (43)	0.004	26 (60)	NA	
Timing of treatments	0	14 (70)	16 (30)	0.47	NA	NA	NA
	1	2 (10)	13 (25)	(0.24; 0.82)	15 (35)	NA	NA
	2	3 (15)	9 (17)	0.005	12 (28)	NA	
	3	1 (5)	15 (28)		16 (37)	NA	
Deaths					14 (47)	6 (14)	0.19 (0.05; 0.64) 0.005
Total		20	53		43	30	

*TPE, Therapeutic plasma exchange*.

*Number of treatments = 0 (no TPE); 1 (1-4 sessions of TPE); 2 (5+ sessions of TPE)*.

*Timing of treatments = days between hospital admission and first TPE: 0 (no TPE); 1 (up to 3 days); 2 (4–5 days); 3 (6–12 days)*.

*Plasmapheresis (TPE) = dichotomous variable which was 0 for 30 untreated patients and 1 for the 29 cured with TPE*.

**Figure 2:**
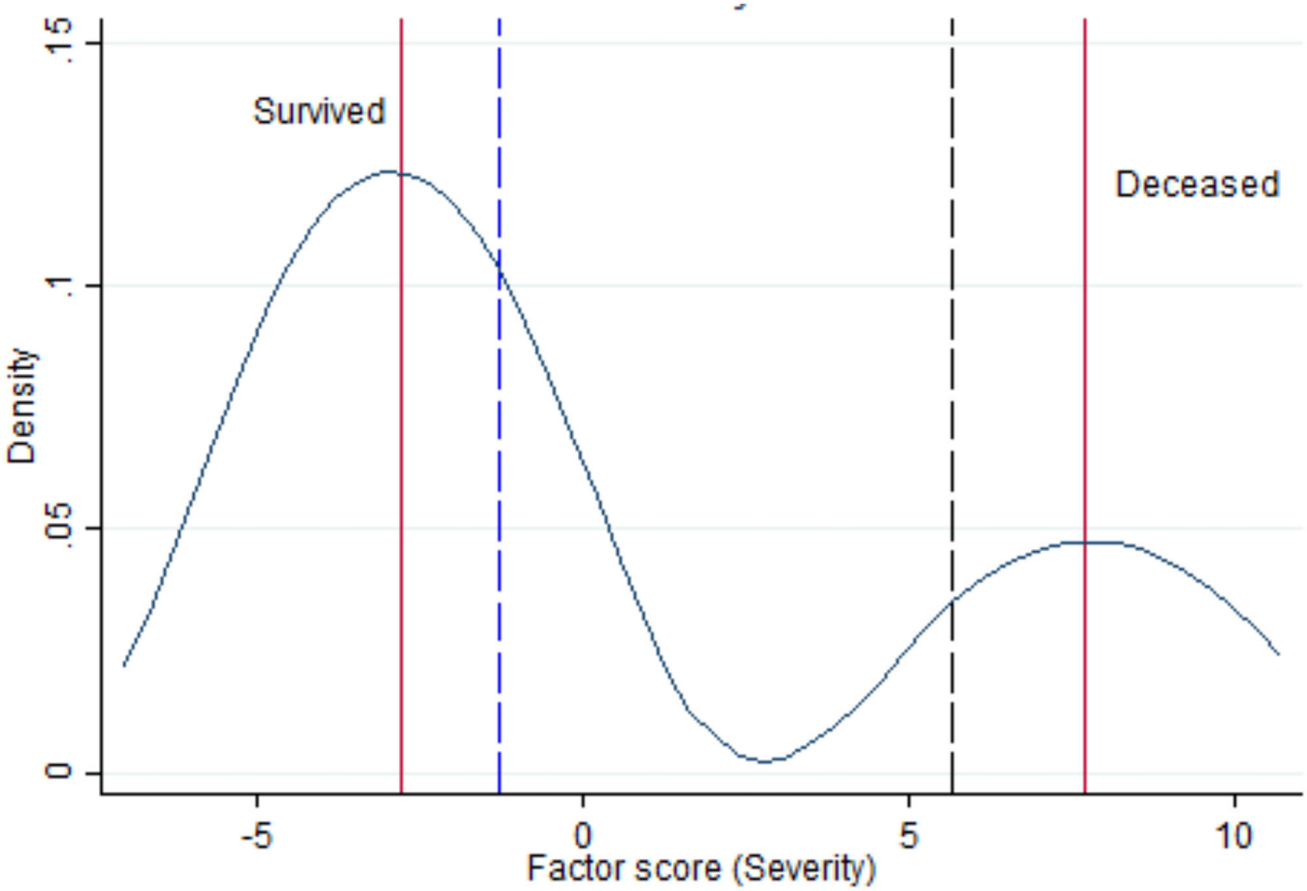
Kernel density estimate for the latent variable “Severity” with vertical lines indicating the median of survived and deceased (red lines), the lowest range of Severity in survivors (blue dashed vertical line) and the highest range of Severity among deceased (black dashed vertical line).

A numerical value of “severity” was estimated by SEM program for each patient. Multiple summary statistics were calculated conditioned on a categorical variable that identified two groups: survived/deceased or TPE-treated/TPE-untreated. The numerical statistics for Severity (min, max, median, 25th, and 75th percentiles) are reported in Table 2, together with the Wilcoxon rank-sum test for the equality of the median distribution across the two groups. The distribution of the Severity score is shown in Figure 2 incorporating various vertical lines to mark the median, min, and max values of the different samples.

**Table 2:** Distribution of the latent variable Severity in 73 patients with COVID-19 by vital status at end of follow-up and by treatment with therapeutic plasma exchange (TPE).

**Severity (arbitrary units)**	**Distribution by vital status**			**Distribution by treatment status**		
	**Deceased** ***N* = 20**	**Survived** ***N* = 53**	**Wilcoxon rank-sum test**	**TPE** ***N* = 43**	**No TPE** ***N* = 30**	**Wilcoxon rank-sum test**
Lowest value	5.66	−5.18		−5.18	−3.89	
Highest value	8.84	−1.27	*z* = −6.555	7.96	8.84	*z* = 4.552
25% percentile	7.24	−3.59	*p* = 0.0000	−3.61	−1.73	*p* = 0.0000
75% percentile	8.49	−2.39		−2.40	8.28	
Median	7.70	−2.80		−2.82	−1.35	

### Statistical Analysis for Assessing TPE Effectiveness

The intervention (TPE) was not randomly allocated to study subjects. To rule out the possibility that any threats to internal validity were responsible for the observed treatment effect, we used three statistical procedures.

#### Propensity Score Matching

The effect of TPE on mortality of patients with COVID-19-associated pneumonia, controlling for sex, personal history of comorbidity, hypertension, diabetes, smoking habit, heaviest oxygen (O_2_) delivery support, and squared age, was estimated by matching the estimated probability of treatment (known as propensity score) and specifying to estimate the average effect on the treated group. After estimation of TPE effect by a matching estimator, a box-plot was produced to check for covariate balance in propensity-score matched sample.

#### Structural Equation Modeling

We used a conceptual framework based on knowledge of the relevant literature contrasting the two central aspects of the study: TPE therapy (including number and timing of administration) and the latent variable severity (estimated using the plausible confounders: sex, comorbidities, hypertension, diabetes, smoking habit, heaviest O_2_ delivery support, and age), using mortality as outcome. All the above assumptions were converted into a SEM model. The effects were expressed as standardized coefficients that make comparisons easier—the higher the coefficient the higher the effect—by ignoring the independent variable's scale of units. We used two goodness-of-fit SEM statistics:

1. the Chi-square test for “model vs. saturated” (the saturated model is the model that fits the covariances perfectly); and
2. the coefficient of determination (CD) that is like *R*^2^ for the whole model, a perfect fit corresponding to a CD of 1. SEM results were both tabulated and presented graphically.

The sample size required for SEM is dependent on model complexity. The best option is to consider the model complexity (i.e., the number of exogenous variables) and the following rules of thumb: minimum ratio 5:1, with a recommended ratio of 10:1, or a recommended ratio of 15:1 for data with no normal distribution (21). With four exogenous variables (TPE, number of treatments, timing of treatments, and severity) used in the SEM model, we should have a minimum of 20 (= 4 × 5) to a maximum of 60 (= 4 × 15) subjects; in total we reached 73 subjects with complete data, thus fulfilling these requirements.

#### Cox Proportional Hazard Models

Furthermore, to examine how the above factors influenced the rate of mortality occurring at a particular point in time, the survival analysis using the Cox proportional-hazards models was adopted. Since the test “rho” of proportional-hazards assumption was not statistically significant for each covariate and the global test was neither statistically significant (data not shown), we started by computing univariable Cox analyses. Then we fitted various models of multivariable Cox analysis to disentangle the joint effect of different factors on patients' survival. In particular:

• One Cox regression model for each of the four covariates: overall TPE, number of TPE treatments, timing of TPE treatments, and disease severity (a numerical value estimated by SEM for each patient).
• Three Cox regression models with two covariates as follows:
  - TPE and disease severity;
  - Number of TPE and disease severity;
  - Timing of TPE treatment and disease severity.
• One Cox regression model with three covariates: number of TPE treatments, timing of TPE treatments, and severity of the disease.

Complete case analysis was adopted including all 73 patients. All analysis were conducted with the statistical package STATA 14.2 (Stata corporaton Lakeway Drive College Station, Texas, USA).

## Results

### Descriptive Results

Under the heading “Distribution by vital status,” Table 1 reports in decreasing order of OR value the risk factors for death from COVID-19 that were observed in our study subjects. O_2_ support was the most important factor associated with death from COVID-19; worthy of notice is that no patient survived invasive mechanical ventilation by intubation. Admission to ICU and hospitalizations longer than 20 days came as second and fourth relevant factors, but they were believed to be collinear variables associated with O_2_ support and no longer considered. Older age increased five times the risk of death (*p* = 0.007). Diabetes and hypertension were found to double the risk of death, although their impact appeared less important probably because of the few cases involved. Comorbidity (other than the above-mentioned disease) appeared to increase only slightly the risk of death. Male sex and smoking did not play any role on mortality. The dichotomous variable “TPE” and the polytomous variables “number of TPE treatments” and “timing of TPE” consistently showed an important protective effect, suggesting TPE as an effective intervention that could protect critically ill COVID-19 cases from death. Data observed in treated and control groups are shown in the Table 1 under the heading “Distribution by treatment status” in the last three columns of the table. It can be seen that whenever an OR in the fourth column was higher than unity, it was lower than unity in the seventh column, suggesting that many harmful exposures (O_2_ support, ICU admission, 65+ years of age, 20+ days of hospitalization, history of diabetes, hypertension, and comorbidities) prevailed in controls instead of being equally distributed between the two groups of treatment. The number and percentage of deaths in the two groups of treatment indicated 14% mortality in the TPE treated group against 47% in the standard-of-care group (OR = 0.19; 95%CI = 0.05; 0.64; *p* = 0.0049). However, the protecting effect of TPE could be due to some confounding characteristics of groups being compared rather than TPE treatment itself.

Low frequencies and sparse data did not allow detecting a meaningful key of interpretation. Therefore, the problem was analyzed with SEM, estimating the latent variable “Severity.” Negative values of the severity score, based on arbitrary units, indicated lower disease severity, whereas positive values suggested higher severity of illness. Table 2 shows that the median severity was 7.70 in survivors and −2.80 in non-survivors, a noteworthy difference (*p* < 0.001). Moreover, the rank-sum test of Wilcoxon proves that the severity medians in treated (= −2.82) vs. untreated (= −1.35) groups were different (*p* < 0.001), even though the respective distributions were overlapping, confirming that treatment assignment involved a selection of patients by initial severity of COVID-19. As can be seen in Figure 2, severity had a bimodal distribution with local maximal values of two modes corresponding to the median value of survived and deceased patients (red vertical lines in left graph). The graph of Figure 2 also shows that the lowest range of severity in survivors (−1.18, blue dashed vertical line) did not overlap with the highest range of Severity among deceased (5.66, black dashed vertical line).

### Outcome Results

#### Propensity Score Matching

Overall, the treatment effect on patients with COVID-19-associated pneumonia receiving TPE was not different from 0 (*p* = 0.855) after controlling for confounding by using matching on the propensity score (data not shown). Therefore, there was no evidence that TPE was effective against mortality of COVID-19-associated pneumonia.

**Figure 3:**
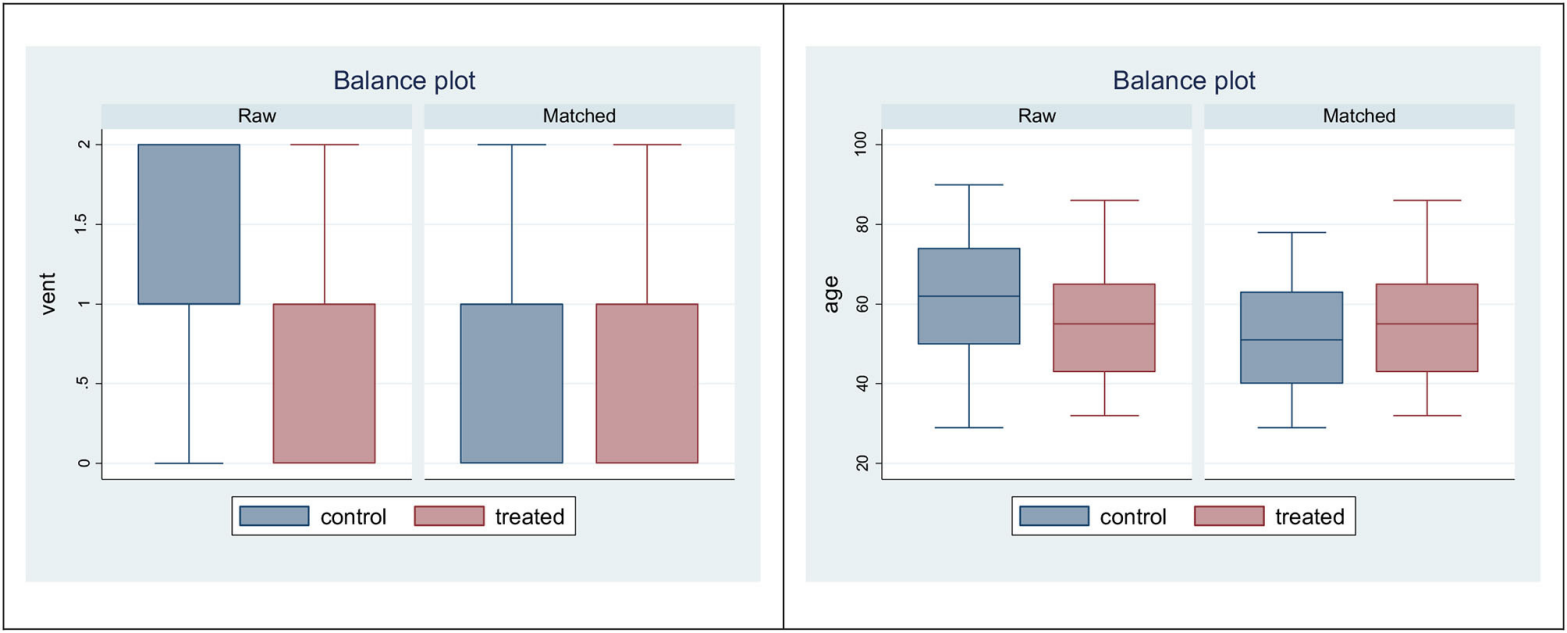
Box plots to check for balance in propensity-score matched sample: variables vent (left) and age (right).

Figure 3 displays the balance plots. In raw data, control subjects had a more severe disease (requiring heavier O_2_ support) and an older age with respect to the treated patients. These differences were reduced after propensity score matching, where matched groups appear to have approximately the same age and the same O_2_ delivery support.

#### Structural Equation Modeling

Table 3 shows the SEM results; it can be seen that death was due to severity of the disease (beta coefficient = 0.94; 95%CI: 0.61–1.27; *p* < 0.001) rather than number or timing of TPE treatment, where effect size was not different from 0. As shown in “Measurement” the latent variable severity was mainly correlated with O_2_ delivery methods (beta coefficient = 0.75; 95%CI: 0.56–0.93; *p* < 0.001) and age (beta coefficient = 0.35; 95%CI: 0.12–0.57; *p* = 0.002), whereas an inconclusive result (*p* = 0.061) was found for diabetes. The findings in the section “Covariances” demonstrated that Severity of disease was negatively correlated with TPE as a whole (beta coefficient = −0.42; 95%CI: −0.69; −0.134; *p* = 0.003); number of treatments (beta coefficient = −0.39; 95%CI: −0.66 to −0.11; *p* = 0.006), and timing of TPE (beta coefficient = −0.40; 95%CI: −0.69 to −0.12; *p* = 0.005), showing that treated subjects had initially a lower severity of disease. The seemingly protective effect of TPE shown in Table 1 was therefore a selection bias.

**Table 3:** Four groups of SEM results (structural equations, measurement, variances, covariances) for the analysis of mortality of critically ill patients with COVID-19 after therapeutic plasma exchange (TPE).

	**Endogenous variables**	**Exogenous variables**	**Beta coef**.	**95% CI**		***P*-value**
				**Lower**	**Upper**	
Structural equation	Deaths	Tpe	0.031	−0.549	0.610	0.917
		N. treatments	−0.074	−0.544	0.395	0.756
		Timing	0.080	−0.308	0.467	0.687
		Severity	0.941	0.614	1.269	0.000
Measurement	Age	Severity	0.348	0.124	0.572	0.002
	Sex	Severity	−0.152	−0.425	0.121	0.276
	Smoking	Severity	−0.058	−0.307	0.192	0.652
	Diabetes	Severity	0.174	−0.065	0.413	0.153
	Co–morbidities	Severity	0.078	−0.180	0.337	0.553
	Hypertension	Severity	0.198	−0.055	0.451	0.124
	Oxygen delivery	Severity	0.745	0.557	0.932	0.000
Variances	var(e.age)	0.879	0.736	1.049	1.05	
	var(e.sex)	0.977	0.897	1.064	1.06	
	var(e.smoking)	0.997	0.968	1.026	1.03	
	var(e.diabetes)	0.970	0.890	1.057	1.06	
	var(e.hypertension)	0.961	0.865	1.066	1.07	
	var(e.co–morbidities)	0.994	0.954	1.035	1.03	
	var(e.oxygen_delivery)	0.446	0.238	0.833	0.83	
	var(e.death)	0.141	0.008	2.461	2.28	
	var(Severity)	1.000	–	–		
Covariances	cov(tpe, Severity)	−0.415	−0.694	−0.137	0.003	
	cov(n_treat, Severity)	−0.385	−0.657	−0.112	0.006	
	cov(timing, Severity)	−0.404	−0.686	−0.121	0.005	

*Standardized beta coefficients (with “minus” sign indicating inverse relationship) with lower and upper limit of 95% confidence intervals (95%CI) and p-values; SEM's goodness-of-fit statistics at the bottom of the table*.

*Goodness of fit statistics:*

*Likelihood ratio test of model vs. saturated: chi2 (38) = 58.31, Prob > chi2 = 0.019*

*Coefficient of determination = 0.884*.

**Figure 4:**
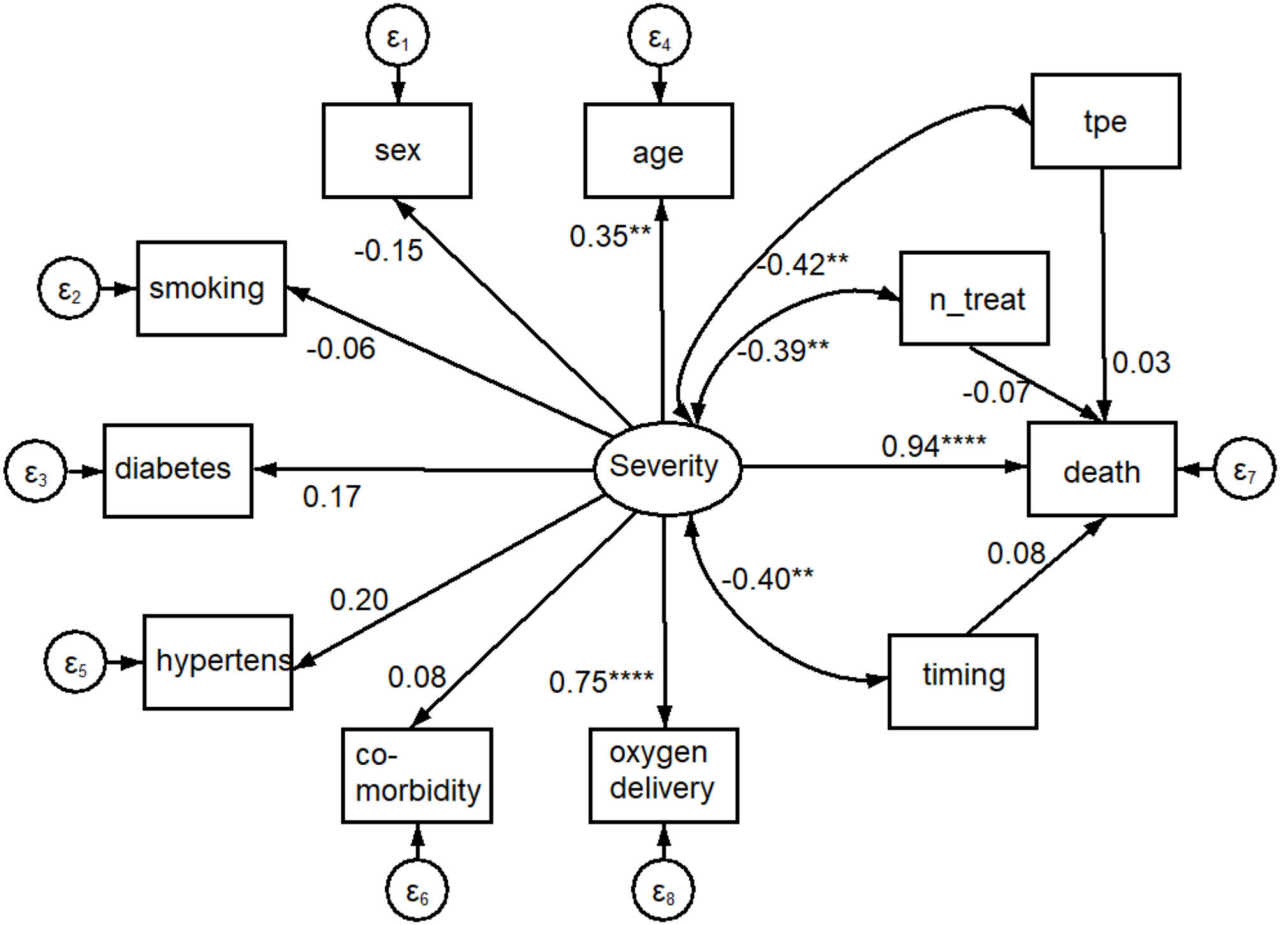
Path diagram of results shown in Table 3. An oval indicates the latent variable (Severity, square boxes indicate the observed variables, circles indicate errors, arrows specify the direction of causal flow, an arrowed route is a path, curved paths express covariances, and the estimated beta coefficients appeared along the paths. It can be seen that: Severity is significantly associated with O_2_ delivery (*p* = 0.000) and age (*p* = 0.002); there is a negative correlation between Severity and plasmapheresis (“tpe,” *p* = 0.003), number (“n_treat,” *p* = 0.006) and timing (“timing,” *p* = 0.005) of its administration; the impact on mortality (the dependent variable “death”) was highest for Severity (*p* = 0.000) and close to 0.0 for plasmapheresis variables. ****p < 0.001, **p < 0.01.

Using the graphical interface of SEM, the same results of Table 3 were displayed as path diagram in Figure 4. In this figure, square boxes stand for variables, arrows specify the direction of causal flow, an arrowed route is a path, and the cross-equation correlation is displayed as a curved path. The estimated beta coefficients with corresponding *p*-values appeared along the paths. The figure is a useful synthesis of the findings. It can be seen that:

- Severity is associated with O_2_ delivery (*p* < 0.001) and age (*p* = 0.002);
- the risk of death was highest for severity (beta coefficient = 0.94; 95%CI: 0.61; 1.27; *p* < 0.001) and close to zero for TPE variables (TPE, number of TPE, and timing of TPE).

#### Cox Proportional Hazard Models

Table 4 shows the results of Cox proportional-hazard models, where the hazard ratios (HR) measure the impact (i.e., the effect size) of covariates. Since its HR was higher than unity (*p* = 0.007), severity decreased the length of survival and was a bad prognostic factor. The univariable HR of 0.68 (95%CI: 0.26; 1.80) for TPE increased to 1.19 (95%CI: 0.43; 3.29) after adjusting for severity. The increasing HR for TPE administration going from univariable to multivariable analyses can be attributed to a confounding effect of disease severity; i.e., the lower mortality observed among patients receiving TPE was due to a lower severity of their disease rather than TPE effects. The same interpretation applies to the parallel changes of the number of TPE and the timing of its administration.

**Table 4:** Effect of therapeutic plasma exchange (TPE) on 30-day mortality of 59 patients hospitalized for severe COVID-19 pneumonia.

**Covariates**	**HR**	**SE**	**95%CI**	***p***
	**Univariate Cox regression analysis**			
Number of TPE treatments	0.71	0.20	(0.41; 1.23)	0.226
Timing of TPE treatments	0.74	0.16	(0.49; 1.13)	0.169
TPE	0.68	0.34	(0.26; 1.80)	0.441
Severity of disease	1.42	0.17	(1.12; 1.79)	0.004
	**Multivariate Cox regression: model with 3 covariates**			
Number of TPE treatments	0.82	0.86	(0.10; 6.39)	0.849
Timing of TPE treatments	1.10	0.94	(0.21; 5.87)	0.910
Severity of disease	1.41	0.18	(1.11; 1.78)	0.005
	**Multivariate Cox regression: models with 2 covariates**			
TPE	1.19	0.62	(0.43; 3.29)	0.741
Severity of disease	1.44	0.19	(1.11; 1.86)	0.006
Number of TPE treatments	0.92	0.27	(0.52; 1.62)	0.767
Severity of disease	1.40	0.17	(1.11; 1.78)	0.005
Timing of TPE treatments	0.94	0.22	(0.59; 1.50)	0.798
Severity of disease	1.41	0.17	(1.11; 1.78)	0.005

*Results of univariate and multivariate Cox proportional-hazards models: hazard ratio (HR), standard error (SE), 95% Confidence Interval (95%CI), and respective p-value (p). One model with 3 and three models with 2 covariates (description in footnote)*.

*Number of treatments: 0 (no TPE); 1 (1–4 sessions of TPE); 2 (5+ sessions of TPE)*.

*Timing of treatments = days between hospital admission and first TPE: 0 (no TPE); 1 (up to 3 days); 2 (4–5 days); 3 (6–12 days)*.

*Severity = latent variable estimated by the STATA program SEM aggregating the variables that affect a worse prognosis of COVID−19 (such as age, sex, smoking, diabetes, hypertension, other comorbidities as well as heaviest oxygen delivery support ever used)*.

*Plasmapheresis = dichotomous variable which was 0 for 30 untreated patients and 1 for the 29 cured with TPE*.

## Discussion

### Key Findings

This single-centered retrospective observational non-placebo-controlled trial enrolled 73 inpatients from Baqiyatallah Hospital in Tehran (Iran) with the diagnosis of COVID-19-associated pneumonia confirmed by RT-qPCR on nasopharyngeal swabs and high-resolution computerized CT scan. These patients were broken down into two groups: Group 1 (30 patients) receiving standard care (corticosteroids, ceftriaxone, azithromycin, pantoprazole, hydroxychloroquine, lopinavir/ritonavir); and Group 2 (43 patients) receiving the above regimen plus TPE (replacing 2 l of patients' plasma by a solution of 50% of normal plasma and 50% of albumin at 5%) administered according to various schedules. The time window of observation was 30 days and all-cause mortality was the endpoint. Deaths were 6 (14%) in Group 2 and 14 (47%) in Group 1.

The intervention (TPE) was not randomly allocated between the two groups of study subjects. To rule out the possibility that any threats were responsible for the observed treatment effect, we used three statistical procedures. First, the effect of treatment was not different from 0 (*p* = 0.855) by using the propensity score matching to control for confounding. Second, the results of SEM showed that death was due to severity of the disease (beta coefficient = 0.94; 95%CI: 0.61–1.27; and *p* < 0.001), rather than number or timing of TPE treatments. Third, the adjustment for confounding was carried out using severity as covariate in Cox regression models. The univariable HR of 0.68 (95%CI: 0.26; 1.80; *p* = 0.441) for TPE increased to 1.19 (95%CI: 0.43; 3.29; *p* = 0.741) after adjusting for severity. Thus, the lower mortality observed among patients receiving TPE was due to a lower severity of their COVID-19 rather than TPE effects.

### Limitations

The lack of randomization is the main limitation of this observational clinical study. Nevertheless, randomizing patients with COVID-19 to receive TPE in the first stages of the pandemic was not feasible due to the emergency situation. However, a comprehensive and advanced statistical analysis was performed to limit the bias associated with the study design.

All patients underwent tests to of creatinine, blood urea nitrogen (BUN), white blood cells (WBC), lymphocytes, neutrophils, hemoglobin (Hb), and platelets on the day of hospital admission and on day 3, day 6, and the last day of hospitalization; whereas levels of c-reactive protein (CRP), lactate dehydrogenase (LDH), creatine phosphokinase (CPK), erythrocyte sedimentation rate (ESR), alanine aminotransferase (ALT), and aspartate transaminase (AST) were assessed only the first and last day of hospital stay. All biological parameters were affected by an excessive number of missing and were never presented in the paper, but can be accessible as Supplementary Tables S1A,1B, S2.

Ferritin, d-dimers, serum il-6 level, SOFA score, and APACHE II score were not available for this study. Therefore, according to a group of physicians from different parts of the world with extensive expertise in clinical apheresis and critical care (22), the diagnosis of CRS could be uncertain. However, in the present study, all 73 patients were affected by severe COVID-19-associated pneumonia with evidence of hypoxemia (respiratory rate > 30/min or partial pressure of O_2_ on arterial blood gas <80 mmHg or PaO_2_/FiO_2_ <300) and lung infiltrates on more than 50% of the lung fields, according to WHO criteria (23).

### Interpretation

The results of the present study indicate 14% mortality in the TPE group against 47% in the standard-of-care only group. However, the risk factors for mortality, such as O_2_ support, ICU admission, 65+ years of age, 20+ days of hospitalization, diabetes, hypertension, and comorbidities, were more common among patients not receiving TPE than those undergoing treatment (Table 1). Summarizing a pool of confounders into a single score by SEM analysis, disease severity was lower among patients with COVID-19 undergoing TPE as compared to control patients (Table 2). Therefore, treatment assignment at hospital admission involved a selection bias in relation to the severity of COVID-19.

While raw data displayed heavier O_2_ support and older age among controls, matched data were similar in the two treatment groups (Figure 3). The comparison with propensity score matching showed that the treatment effect was not different from zero (data not shown). SEM structural equation modeling analysis showed that mortality was explained by disease severity, rather than TPE or number of TPE or timing between date of TPE administration and date of hospital admission (Table 3). Cox-regression models, which were carried out using severity as covariate rather than a large number of measured pretreatment covariates to avoid over-fitting and collinearity issues, confirmed that the lower mortality among patients receiving TPE was due to a lower severity of their disease, not to TPE effects nor to the number of TPE and timing of its administration (Table 4).

Overall, our results consistently evidence that patients receiving TPE were affected by less severe COVID-19-associated pneumonia, and TPE was not effective to prevent the relative mortality.

### Generalizability

As shown in Table 3, severity was mostly correlated with maximum O_2_ delivery support (beta coefficient = 0.75; 95%CI = 0.56–0.93); *p* < 0.001). Analyzing the clinical characteristics of survivors vs. non-survivors of COVID-19 in Wuhan (China), Deng et al. (24) found a lower O_2_ saturation level (*Z* = 10.625; *p* < 0.001) among non-survivors (O_2_ sat: 85%; range: 77–91%) compared to survivors (O_2_ sat: 97%; range: 95–98%). Another striking finding was that the lowest range of O_2_ saturation range in survivors (95%) did not overlap with the highest range of O_2_ saturation among non-survivors (91%) (24). The latter findings agree with those of Figure 2 of the present study, showing that the lowest range of Severity in survivors (−1.18, blue dashed vertical line) did not overlap with the highest range of Severity among deceased (5.66, black dashed vertical line).

It has been reported that in patients affected by severe COVID-19, the cytokine storm was higher around 7–14 days after the disease onset (25). Thus, timely initiation of TPE within this period could determine better patient's outcomes. Additionally, TPE needs to be administered for the correct duration and quantity (26). The effect of TPE in sepsis has shown that both the timing and disease severity are important for the beneficial effect of the procedure (27). Therefore, we coded two variables, one accounting for the number of treatments administered and another for timing of the first administration. Along with TPE treatment, the latter two variables were included in SEM analysis (Table 3) and Cox regression models (Table 4). All of them showed no effect neither on mortality (Table 3) nor on survival (Table 4).

The current evidence on the effect of TPE in severe/critical COVID-19 was summarized by Lu et al. (28). Among the 24 studies reviewed, 21 had a nonexperimental design (case-reports or case-series without controls), in which the effectiveness of the intervention had been assessed by before-and-after comparison of findings. The before-and-after design must be used with caution because several circumstances, called threats to internal validity, bias the ability to correctly infer whether the intervention had the desired effect. Some of these studies (29–33) focused COVID-19-associated pneumonia or COVID-19 related acute respiratory distress syndrome (ARDS) as endpoints, and TPE as the treatment. TPE protocols were quite heterogenous, the treatment schedules ranging from one to nine procedures, usually administered daily, but sometimes every other day. Fresh frozen plasma was specified as the replacement fluid for many, but not all studies. The duration of the procedure and plasma volume exchanged were also variable. TPE with replacement of convalescent plasma was suggested by Kesici et al. (34) as an early treatment option for patients with severe COVID-19, within the first week of symptom onset. Tabibi et al. (35) recommended further exploring TPE by clinical trials before using it in management of COVID-19-induced ARDS and multiorgan dysfunction. On the basis of these studies, no conclusions on TPE as a potential treatment for SARS-CoV-2 infection can be drawn.

According to Honore patients in a deadly proinflammatory state or in an antiinflammatory state that could help them to survive are difficult to be differentiated (36). Therefore, it is impossible to know whether an indiscriminate TPE will remove detrimental proinflammatory mediators in excess (e.g., IL-6) or protective anti-inflammatory proteins (e.g., antibodies, complement component 3 and 4) that may support patient's survival (36). TPE might remove critically important neutralizing antibodies against SARS-CoV-2 regardless of the inflammatory state of the patient, as confirmed by a recent case of life-threatening COVID-19–associated sepsis, with anti SARS-CoV-2-specific IgG and IgA antibodies found in the plasma bag following TPE and reduction by one log step of patient's circulating antibodies (37). Furthermore, the reduction of serum inflammatory mediators by TPE might produce a dilution of those molecules, not necessarily translating into an improvement of the septic status of the patient, who may need additional treatments (38).

Out of the 24 studies reviewed by Lu et al. (28), 3 had a quasi-experimental design, using two groups of COVID-19 patients, either treated or untreated with TPE, to disentangle the effects of TPE from those of additional treatments. No difference in all-cause mortality was reported by two (39, 40) out of three studies. The third study (41) is discussed below together with a similar study (42) found by us.

In both the latter studies, treatment assignment was a non-random procedure and pretreatment characteristics of groups were not comparable. Nonetheless, direct comparison between treated and untreated groups was carried out, finding an unconvincing difference in mortality (*p* = 0.037) in the study by Gucyetmez et al. (41) and in time for CRS resolution (*p* = 0.04) in the study by Kamran et al. (42). These findings could be explained by either the treatment or pretreatment variables, or a combination of both. To form matched groups of treated and untreated individuals with similar or comparable pretreatment characteristics, the propensity score matching (PSM) was applied to both Gucyetmez et al. (41) and Kamran et al. (42) studies. After PSM, the two matched groups (TPE and non-TPE), each composed of 45 patients (36, 42) or 12 patients (41), had comparable pretreatment characteristics, and hence differences in the respective outcome became more significant (*p* = 0.001 in the former and *p* = 0.009 in the latter study). However, while in RCT randomization ensures comparability in both measured and unmeasured pretreatment characteristics, in PSM the comparability of the treatment groups is limited only to measured pretreatment characteristics included in the propensity score model. In Gucyetmez's study (41), there were 18 patients treated and 35 patients untreated, becoming 12 in each group after PSM. In the Kamran's study (42) the corresponding figures were 71 (TPE), 209 (non-TPE), and 45 in each group after PSM. During matching, the closest untreated and treated individuals are matched and the remaining individuals that were not matched are excluded from the analysis. Exclusion of unmatched individuals from the analysis not only affects the precision of the treatment effect estimates but also could have consequences for the generalizability of the findings (43).

The last study is a prospective RCT (5); hence with experimental design, based on the comparison of 43 TPE treated patients with 44 patients receiving only the standard of care, all randomly selected among patients with COVID-19 admitted to ICU. Primary outcomes were mortality at 35 days post ICU admission and safety of TPE in life-threatening COVID-19. The multivariable Cox proportional hazards model showed no significant effect of TPE on 35-day survival after adjustment for relevant confounders, therefore aligning with the results of the present study.

Studies have suggested that in about 10% of cases, high levels of preexisting autoantibodies (auto-Abs) neutralizing type I interferon (IFN), remove type I IFN–dependent control of SARS-CoV-2 replication *in vitro*. As the risk of developing severe, and even potentially fatal, COVID-19-associated pneumonia is extremely high in patients harboring auto-Abs against type I IFN, eliminating these auto-Abs by TPE emerges as an attractive additional line of treatment. However, a prospective RCT is required to better define its use (44), including the number of sessions needed and choice of volume replacement as additional factors in the analysis. The feasibility of this approach is contingent on the availability of a certified assay to detect and quantify neutralizing auto-Abs to type I IFN (44).

## Conclusions

The lower mortality observed among patients with COVID-19 receiving TPE was due to a lower severity of the disease rather than TPE effects. However, this finding still does not rule out the potential benefit of TPE for those patients with circulating autoantibodies against Interferon type 1, which deserves further investigation in more focused RCT and prior detection/dosage of autoantibodies.

## Data Availability Statement

The raw data supporting the conclusions of this article will be made available by the authors, without undue reservation.

## Ethics Statement

This study involving human participants was reviewed and approved by the Ethics Committee of Baqiyatallah University of Medical Science (Tehran, Iran); trial registration number: IRCT20080901001165N58 (Iranian Registry of Clinical Trials); Registration date: 2020-05-27, 1399/03/07 (retrospectively registered). The patients/participants provided their written informed consent to participate to this study.

## Author Contributions

LC and GM designed and ran the analysis, interpreted the data, and drafted the original draft. BE, YP, MJ, SI, MR, MN, and HA conceived the study, collected the data, contributed to interpret the data, and validated the manuscript. All authors contributed to the article and approved the submitted version.

## Conflict of Interest

The authors declare that the research was conducted in the absence of any commercial or financial relationships that could be construed as a potential conflict of interest.

## Publisher's Note

All claims expressed in this article are solely those of the authors and do not necessarily represent those of their affiliated organizations, or those of the publisher, the editors and the reviewers. Any product that may be evaluated in this article, or claim that may be made by its manufacturer, is not guaranteed or endorsed by the publisher.
