# Supplementary material for: On Therapeutic Plasma Exchange Against Severe COVID-19-Associated Pneumonia: An Observational Clinical Study
Source: Front Nutr. 2022 Feb 22;9:809823. doi: 10.3389/fnut.2022.809823 (PMC8926159; doi:10.3389/fnut.2022.809823)
Supplement: Supplementary file 1 [file Table_1.docx]

| **Supplementary Table S1a. Patients not treated with Therapeutic Plasma Exchange (TPE).** Distribution of clinical parameters over time, from hospital admission until end of follow-up (hospital discharge or death). Hb=Haemoglobin; BUN= Blood Urea Nitrogen; CRP= C Reactive Protein; LDH= lactate dehydrogenase; CPK= Creatine Phosphokinase; ESR= Erythrocyte Sedimentation Rate; ALT= Alanine Aminotransferase; AST= Aspartate aminotrasferase; CT score= Computerized tomography score; NEWS= New Early Warning Score. | | | | | | | | |
| --- | --- | --- | --- | --- | --- | --- | --- | --- |
| **Parameter** | | **Admission (base)** | **Day 3*** | **p value** | **Day 6*** | **p value** | **Final*** | **p value** |
| WBC  (10^9^ cells /L) | Mean ± SD | 9.81 ± 7.04 | -0.33 ± 5.66 | 0.379 | 10.09 ± 6.61 | 0.192 | 1.68 ± 8.23 | 0.137 |
|  | Median (IQ) | 7.8 (5.2; 11.2) | 0.3 (-1.5; 2.7) |  | 2.2 (-1.4; 5.2) |  | 0.6 (-1.7; -6.3) |  |
| Neutrophils  (cells/µL) | Mean ± SD | 7,175.09 ± 4.418.85 | 1,337.17 ± 3,396.98 | 0.057 | 2.304;16 ± 3.845.90 | **0.009** | 3,167.74 ± 7,094.77 | 0.034 |
|  | Median (IQ) | 6.630.0 (4.160.0; 8.415.0) | 1,524.5 (3.0; 3029.0) |  | 2,494.0 (-1,195.0; 5,695.0) |  | 47.0 (-1,711.0; 7.650.0) |  |
| Lymphocytes  (10^9^ cells /L) | Mean ± SD | 1,136.73 ± 705.69 | -232.90 ± 466.84 | **<0.001** | -53.61 ± 768.57 | 0.369 | 159.86 ± 837.88 | 0.844 |
|  | Median (IQ) | 957.5 (538.0; 1,576.0) | 295.0 (-586.0; 65.0) |  | 107 (-648.5; 458.5) |  | 157.0 (-527.0; 719.0) |  |
| Hb  (g/dl) | Mean ± SD | 13.47 ± 2.07 | -1.41 ± 0.99 | **<0.001** | -1.66 ± 1.97 | **<0.001** | -2.53 ± 2.15 | **<0.001** |
|  | Median (IQ) | 13.4 (12.8; 14.4) | -1.4 (-1.8; -0.8) |  | -1.7 (-2.4; -0.2) |  | -2.5 (-3.6; -1.1) |  |
| Platelets  (10^9^ cells /L) | Mean ± SD | 202.93 ± 87.00 | 23.92 ± 70.29 | 0.051 | 56.64 ± 129.58 | **0.014** | 23.18 ± 148.12 | 0.208 |
|  | Median (IQ) | 190.0 (151.0; 211.0) | 24.0 (-20.0; 53.0) |  | 34.5 (-16.5; 1.06.0) |  | 12.0 (-93.5; 119.0) |  |
| Creatinine  (mg/dL) | Mean ± SD | 1.55 ± 1.76 | -0.01 ± 0.87 | 0.483 | -0.0 ± 1.3 | 0.494 | 0.21 ± 1.08 | 0.164 |
|  | Median (IQ) | 1.1 (0.8; 1.6) | -0.1 (-0.3; 0.1) |  | -0.1 (-0.2; 0.3) |  | 0.0 (-0.3; 0.5) |  |
| BUN  (mg/dL) | Mean ± SD | 23.63 ± 17.45 | 3.38 ± 10.92 | 0.053 | 7.79 ± 14.30 | **0.004** | 12.54 ± 22.74 | **0.005** |
|  | Median (IQ) | 17.0 (12.0; 30.0) | -3.0 (-0.1; 6.0) |  | 6.0 (0.0; 19.0) |  | 9.0 (0.0; 24.0) |  |
| CRP  (mg/L) | Mean ± SD | 36.78 ± 40.47 |  |  |  |  | -30.62 ± 41.15 | **0.010** |
|  | Median (IQ) | 22.8 (11.2; 50.0) |  |  |  |  | -7.6 (-64.5; -2.2) |  |
| LDH  (U/L) | Mean ± SD | 883.81 ± 349.88 |  |  |  |  | 145.18 ± 457.76 | 0.159 |
|  | Median (IQ) | 819.0 (712.0; 1,066.0) |  |  |  |  | 182.0 (-98.0; 266.0) |  |
| CPK  (Units/L) | Mean ± SD | 421.31 ± 593.47 |  |  |  |  | 0 | NA |
|  | Median (IQ) | 170.0 (124.5; 516.0) |  |  |  |  | 0 |  |
| ESR  (mm/hour) | Mean ± SD | 60.07 ± 32.02 |  |  |  |  | -7.20 ± 33.31 | 0.327 |
|  | Median (IQ) | 54.0 (34.0; 83.5) |  |  |  |  | 0 (-39.5; 21.5) |  |
| ALT  (IU/L) | Mean ± SD | 54.74 ± 38.23 |  |  |  |  | 3.20 ± 51.50 | 0.424 |
|  | Median (IQ) | 45.0 (25.0; 79.0) |  |  |  |  | 20.0 (-38.5; 42.5) |  |
| AST  (IU/L)) | Mean ± SD | 48.48 ± 33.88 |  |  |  |  | 7.70 ± 40.52 | 0.281 |
|  | Median (IQ) | 37.0 (28.0; 56.0) |  |  |  |  | 2.0 (-7.3; 16.5) |  |
| CT score | Mean ± SD | 17.70 ± 3.43 |  |  |  |  | -5.22 ± 7.57 | **0.005** |
|  | Median (IQ) | 18.0 (15.0; 21.0) |  |  |  |  | -5.0 (-10.0; 2.0) |  |
| NEWS score | Mean ± SD | 5.57 ± 1.70 |  |  | 1.07 ± 2.09 | **0.034** | 0.74 ± 5.77 | **0.255** |
|  | Median (IQ) | 6.0 (4.8; 7.0) |  |  | -1.0 (-2.8; 1.8) |  | -2.0 (-4.0; 6.0) |  |

- Difference between day3 and baseline estimate

** Difference between day6 and baseline estimate

*** Difference between final and baseline estimate

| **Supplementary Table S1b. Patients treated with Therapeutic Plasma Exchange (TPE).** Distribution of clinical parameters over time, from hospital admission until end of follow-up (hospital discharge or death). Hb=Haemoglobin; BUN= Blood Urea Nitrogen; CRP= C Reactive Protein; LDH= lactate dehydrogenase; CPK= Creatine Phosphokinase; ESR= Erythrocyte Sedimentation Rate; ALT= Alanine Aminotransferase; AST= Aspartate aminotransferase; CT score= Computerized tomography score; NEWS= New Early Warning Score. | | | | | | | | |
| --- | --- | --- | --- | --- | --- | --- | --- | --- |
| **Parameter** | | **Admission (base)** | **Day 3*** | **p value** | **Day 6**** | **p value** | **Final**** | **p value** |
| WBC  (10^9^ cells /L) | Mean ± SD | 8.85 ± 4.86 | 3.07 ± 4.35 | **<0.001** | 4.49 ± 4.04 | **<0.001** | -2.78 ± 4.94 | **0.001** |
|  | Median (IQ) | 7.3 (4.9-12.7) | 2.9 (6.1; 0.1) |  | 4.9 (7.3; 1.8) |  | -3.2 (-7.2; -0.4) |  |
| Neutrophils  (cells/µL) | Mean ± SD | 7.456.88 ± 4,748.86 | 2,940.83 ± 4,201.52 | **<0.001** | 4,325.24 ± 3,149.85 | **<0.001** | -2,303.06 ± 4,815.53 | **0.005** |
|  | Median (IQ) | 5,808 (3,885; 10,528) | 3,746.0 (0; 5,118.0) |  | 5,204 (6,490.0; 1,739.0) |  | -2,879.0 (-6,343.0; 381.0) |  |
| Lymphocytes  (10^9^ cells /L) | Mean ± SD | 1,017.59 ± 643.96 | -228.13 ± 823.13 | 0.064 | -96.73 ± 1,074.03 | 0.325 | -272.66 ± 933.57 | 0.047 |
|  | Median (IQ) | 802.0 (637.0; 1,234.0) | -111.9 (-527.5; 189.0) |  | 62.0 (-405.0; 700.0;) |  | 306.0 (-252.0; 757.0) |  |
| Hb  (g/dl) | Mean ± SD | 13.65 ± 2.19 | 1.23 ± 1.42 | **<0.001** | -2.22 ± 2.30 | **<0.001** | -2.39 ± 2.97 | **<0.001** |
|  | Median (IQ) | 13.5 (12.4; 14.7) | -1.5 (-2.3; -0.3) |  | -1.60 (-3.60; -0.70) |  | -2.4 (-4.5; -0.8) |  |
| Platelets  (10^9^ cells /L) | Mean ± SD | 211.65 ± 95.97 | 37.81 ± 71.73 | **0.003** | 19.29 ± 108.12 | 0.158 | -7.00 ± 108.88 | 0.353 |
|  | Median (IQ) | 199.0 (143.0; 258.0) | 36.0 (-16.0; 97.0) |  | -4.5 (-57.5; 84.5) |  | 1.0 (-56.0; 107.0) |  |
| Creatinine  (mg/dL) | Mean ± SD | 1.50 ± 2.72 | -0.42 ± 2.08 | 0.154 | 0.34 ± 2.17 | 0.231 | 0.38 ± 2.11 | 0.286 |
|  | Median (IQ) | 1 (0.9; 1.1) | 0.0 (-1.0; 0.0) |  | 0.10 (-0.10; 0.20) |  | -0.1 (-0.3; 0.0) |  |
| BUN  (mg/dL) | Mean ± SD | 19.67 ± 13.48 | 4.03 ± 9.51 | **0.015** | 4.43 ± 13.68 | 0.043 | -4.91 ± 16.74 | 0.046 |
|  | Median (IQ) | 16.5 (11.0; 22.0) | 4.0 (1.0; 8.0) |  | -0.1 (-0.2; 0.1) |  | 6.0 (-1.0; 12.0) |  |
| CRP  (mg/L) | Mean ± SD | 22.31 ± 12.07 |  |  |  |  | -0.38 ± 26.47 | 0.476 |
|  | Median (IQ) | 21.1 (15.6; 28.4) |  |  |  |  | -8.3 (-15.8; 7.1) |  |
| LDH  (U/L) | Mean ± SD | 785.08 ± 251.14 |  |  |  |  | 24.82 ± 1,054.45 | 0.462 |
|  | Median (IQ) | 697.0 (606.0; 914.0) |  |  |  |  | -196.0 (-278.0;- 93.0) |  |
| CPK  (Units/L) | Mean ± SD | 180.23 ± 107.67 |  |  |  |  | -20.71 ± 57.18 | 0.187 |
|  | Median (IQ) | 155.5 (99.0; 236.0) |  |  |  |  | -29.0 (-65.0; 21.0) |  |
| ESR  (mm/hour) | Mean ± SD | 53.63 ± 30.78 |  |  |  |  | -21.51 ± 40.75 | 0.035 |
|  | Median (IQ) | 47.0 (34.0; 70.5) |  |  |  |  | 22.6 (-56.0; 9.0) |  |
| ALT  (IU/L) | Mean ± SD | 95.26 ± 45.28 |  |  |  |  | -12.39 ± 31.79 | 0.386 |
|  | Median (IQ) | 97.5 (71.0; 115.0) |  |  |  |  | -10.0 (-30.0; 17.0) |  |
| AST  (IU/L)) | Mean ± SD | 47.26 ± 28.57 |  |  |  |  | -4.21 ± 62.61 | 0.058 |
|  | Median (IQ) | 41.5 (27.0; 55.0) |  |  |  |  | -9.0 (-52.0; 24.0) |  |
| CT score | Mean ± SD | 17.95 ± 3.82 |  |  |  |  | -10.14 ± 4.53 | **<0.001** |
|  | Median (IQ) | 19.0 (16.5; 21.0) |  |  |  |  | -10.0 (-14.0; -7.0) |  |
| NEWS score | Mean ± SD | 5.79 ± 2.22 |  |  | -1.37 ± 3.21 | **0.005** | 2.97 ± 4.91 | **<0.001** |
|  | Median (IQ) | 6.0 (4.0; 7.0) |  |  | -1.0 (-4.0; 0) |  | -4.0 (-6.0; -2.0) |  |

- Difference between day3 and baseline estimate

** Difference between day6 and baseline estimate

*** Difference between final and baseline estimate

| **Supplementary Table S2.** Number and average missing values of biologic parameters over time. NA= not available. WBC= White Blood Cells; Hb=Haemoglobin; BUN=Blood Urea Nitrogen; CRP= C Reactive Protein; LDH= lactate dehydrogenase; CPK= Creatine Phosphokinase; ESR= Erythrocyte Sedimentation Rate; ALT= Alanine Aminotransferase; AST= Aspartate aminotransferase. | | | | | |
| --- | --- | --- | --- | --- | --- |
| **PARAMETER** | **ADMISSION** | **DAY 3** | **DAY 6** | **FINAL** | **AVERAGE** |
| WBC | 0 | 11 | 13 | 6 | 7.5 |
| Neutrophils | 10 | 21 | 23 | 17 | 17.8 |
| Lymphocytes | 2 | 11 | 17 | 7 | 9.3 |
| Hb | 0 | 11 | 13 | 9 | 8.3 |
| Platelets | 2 | 15 | 15 | 8 | 10.0 |
| Creatinine | 1 | 14 | 15 | 11 | 10.3 |
| BUN | 1 | 14 | 15 | 11 | 10.3 |
| **AVERAGE** | **2.3** | **13.9** | **15.9** | **9.9** | **10.5** |
| CRP | 9 | NA | NA | 39 | 24 |
| LDH | 10 | NA | NA | 43 | 26.5 |
| CPK | 27 | NA | NA | 64 | 45.5 |
| ESR | 5 | NA | NA | 53 | 29 |
| ALT | 12 | NA | NA | 43 | 27.5 |
| AST | 12 | NA | NA | 44 | 28 |
| **AVERAGE** | **12.5** |  |  | **47.7** | **30.1** |
